# Statistics of the Lying-in Hospital at Berlin

**Published:** 1853-05

**Authors:** 


*Statistics of the Lying-in Hospital at Berlin.*—The number of accouchements from 1st January, 1836, till 31st December, 1841, was 4124, of which 1435 were *primiparæ.* The number of women delivered in the hospital was 1349, of whom 27 died. The number delivered in their own houses was 2775, of whom 33 died. The number of twin cases was 58. Of the 4182 children, 2141 were males, 1997 females, and of 44 the sex is not given. Of 4080 born at the full time, 262 were still-born; of these 56 were in the hospital, and 206 in the patients' houses. 102 were born before the full period. Presentations—of head 3846, face 26, brow 3, breech 98, footling 63, knee 2, 83 completely irregular. In 61 cases of premature labor the presentation could not be discovered. 3572 deliveries took place without assistance; while in 552 the following assistance was required:—In 358 the forceps were applied; 101 extractions of the child; 2 irregular presentations rectified; 14 turning on the head, 1 on the breech, 75 on the feet; 7 forced deliveries, 7 premature deliveries; 6 cases of perforation; 8 of breaking down the head; 1 embryotomy; 2 cæsarian operations during the life of the mother, 1 after death; in 9 cases the cord, having prolapsed alongside of the head, was replaced; the placenta was artificially removed in 110 cases; in 3 the perineum was incised.

There were 67 cases of contraction of the pelvis from rickets. Of these, delivery was effected in 3 by the cæsarian section, in 1 by embryotomy, in 5 by perforation, in 8 by breaking down the head, in 5 by inducing premature labor, in 41 by the forceps, and in 2 by bringing down the feet.

Of the above 67, 9 mothers died; 2 after the cæsarian operation, 1 after embryotomy, 1 after perforation, 1 after breaking down the head, 2 after difficult delivery by the forceps, and 2 of hæmorrhage, following in one case, perforation, in the other, the application of the forceps.

In these cases 36 children were delivered dead, 32 (1 case of twins) were delivered alive.

Of the children born dead 5 were delivered by perforation, 8 by breaking down the head, 1 by embryotomy, 1 by artificial premature birth, 5 by turning and extraction by the feet, 1 by delivery by the feet, 14 by application of the forceps, and 1 by the cæsarian operation.—*Glasgow Med. Journ. from A. Lereboullet und M. Ruefz. Zeitschrift fur Geburtskunde.*

